# Systematic Review of the Effects of Plant-Based Foods on Metabolic Outcomes in Adults with MASLD and Comorbidities Such as Obesity, Metabolic Syndrome, and Type 2 Diabetes

**DOI:** 10.3390/nu17183020

**Published:** 2025-09-22

**Authors:** Joanna Michalina Jurek, Katarzyna Zablocka-Slowinska, Joanna Pieczynska, Helena Clavero Mestres, Teresa Auguet

**Affiliations:** 1 Grup de Recerca GEMMAIR (AGAUR)-Medicina Aplicada (URV), Departament de Medicina i Cirurgia, Universitat Rovira i Virgili (URV), Institut d’Investigació Sanitària Pere Virgili (IISPV), Mallafré Guasch, 4, 43007 Tarragona, Spain; joanna.michalina.jurek@gmail.com (J.M.J.);; 2 The Faculty of Finance and Management, WSB Merito University Wroclaw, Fabryczna 29/31, 53-609 Wroclaw, Poland; katarzyna.zablocka-slowinska@wroclaw.merito.pl; 3 Department of Dietetics and Bromatology, Wroclaw Medical University, Borowska 211, 50-556 Wrocław, Poland; joanna.pieczynska@umw.edu.pl; 4 Servei Medicina Interna, Hospital Universitari de Tarragona Joan XXIII, Mallafré Guasch, 4, 43007 Tarragona, Spain

**Keywords:** plant foods, metabolic outcomes, obesity, MASLD/non-alcoholic fatty liver disease, type 2 diabetes, metabolic syndrome, systematic review

## Abstract

**Background**: Metabolic dysfunction-associated steatotic liver disease (MASLD) has become one of the most prevalent liver diseases, affecting up to 40% of adults and strongly associated with obesity and metabolic dysfunction. Despite the lack of approved pharmacological treatments, dietary interventions with plant-based foods, including the Mediterranean diet (MED), rich in numerous bioactive compounds may offer benefits for metabolic health and hepatic function. However, the role of individual plant foods in MASLD management remains unclear. **Objectives**: This review investigates the effects of specific plant-based foods, consumed as part of the MED and Dietary Approaches to Stop Hypertension (DASHs) diet, on metabolic outcomes, including hepatic function, in MASLD patients alone or in combination with comorbidities such as obesity, metabolic syndrome, and type 2 diabetes mellitus (T2DM). **Methods**: A systematic search was registered and conducted across nine databases to identify randomized controlled trials (RCTs) carried out in adults with MASLD and published between January 2020 and May 2025, following PRISMA guidelines. **Results**: Plant-based interventions including oranges, whole-grain products (WGPs), high-fiber buns (HFBs), beetroot juice (BJ), garlic, ginger, flaxseed, spirulina, rapeseed oil, sour tea, and green coffee extract (GCE) demonstrated mixed effects on metabolic and hepatic outcomes. GCE, flaxseed, and rapeseed oil improved anthropometric measures, while sour tea and ginger supported blood pressure control. WGPs, GCE, flaxseed, rapeseed oil, spirulina, ginger, and garlic were beneficial for glycemic regulation, whereas WGPs, HFBs, BJ, golden flaxseed, rapeseed oil, and garlic improved lipid profiles. Liver enzymes improved following consumption of WGPs, BJ, sour tea, flaxseed oil, and garlic, and hepatic steatosis was reduced after intake of oranges, WGPs, HFBs, BJ, flaxseed powder, rapeseed oil, and garlic powder. Conversely, a solely fruit-rich diet (FRD) had negative effects across all outcomes. **Conclusions**: Plant-based foods improved metabolic outcomes, with WGPs, HFBs, beetroot, oranges, sour tea, flaxseed oil, and garlic providing specific benefits for liver health. Further research is needed to validate these effects and ensure their safety in MASLD management.

## 1. Introduction

Metabolic dysfunction-associated steatotic liver disease (MASLD), previously referred to as non-alcoholic fatty liver disease (NAFLD), has emerged as a widespread chronic condition, currently estimated to affect up to 40% of the adult population worldwide [1,2]. MASLD encompasses a broad continuum of hepatic abnormalities, ranging from simple steatosis (SS), defined as lipid accumulation in more than 5% of hepatocytes, to metabolic dysfunction-associated steatohepatitis (MASH), progressive fibrosis, cirrhosis, and hepatocellular carcinoma (HCC) [3]. These pathological manifestations are primarily driven by excessive triglyceride deposition within hepatocytes, in conjunction with at least one cardiometabolic risk factor, most notably obesity, type 2 diabetes mellitus (T2DM), and dyslipidemia, which represent the most prevalent and clinically relevant contributors [1,4].

Visceral adiposity and chronic low-grade inflammation act synergistically to promote hepatic lipid infiltration and oxidative stress, while insulin resistance and dysregulated lipid metabolism exacerbate intrahepatic injury [5,6]. In addition, T2DM, reported in 30–60% of MASLD patients, is considered a key risk factor for steatosis and driver of cirrhosis [7]. Similarly, persistent dyslipidemia was reported as a potential contributor to increased lipotoxicity and oxidative stress in hepatocytes that promotes inflammation and fibrogenesis [8]. All together, these overlapping metabolic disturbances support the association between MASLD and increased cardiovascular and renal morbidity and mortality [2]. Despite the high burden of MASLD, to date, there are no pharmacological therapies formally approved for this condition, apart from the selective oral thyroid hormone receptor beta agonist Resmetirom, which was approved for the treatment of non-cirrhotic MASH with moderate-to-advanced fibrosis in the USA [9]. Additionally, since August 2025 Food and Drug Administration (FDA) has approved semaglutide (WEGOVY) injection with a weekly dose of 2.4 mg as treatment for metabolic-associated steatohepatitis (MASH) with moderate-to advanced fibrosis [9]. Therefore, the official clinical guidelines [1] recommending a combined dietary and lifestyle approach [10,11] remain the basic treatment option for all phases of the disease. These modifications, including both calorie restriction within the Mediterranean diet (MED) and increased physical activity (≥150 min/week of moderate intensity), along with treatment of underlying risk factors [1], have been shown to be effective in body weight reduction, which is associated with a significant improvement in histological outcomes of MASH, including a reduction in hepatic steatosis and fibrosis [12]. In addition, modification of the macronutrient composition, either by a low-carbohydrate diet or MED, has demonstrated similar effectiveness in decreasing body weight and blood pressure as well as levels of glucose, insulin, and hepatic transaminases in MASLD [13].

Maintaining restrictive or intensive lifestyle changes, along with the achieved body weight, remains challenging in clinical practice, which has led to a growing interest in novel strategies to support liver and metabolic health. Although various nutritional modifications have been investigated, including adjustments in macronutrient composition [13,14] and food quantity and quality [15,16], as well as time-restricted eating [17], different models of plant-based diets (PBDs) [14,18] appear to play an important role in the long-term management of MASLD. These dietary patterns, characterized by high intakes of vegetables, fruits, legumes, whole grains, nuts, and unsaturated fats [18,19,20], seem to be particularly effective in reducing anthropometric measures, such as body weight, body mass index, and waist circumference, as well as improving glycemic control and inflammation [18,19,20]. Greater adherence to PBDs, such as the Mediterranean diet (MED), has been shown to significantly reduce the risk of steatotic liver disease (SLD), MASLD, and alcohol-related liver diseases (ALDs), suggesting that following this dietary pattern may confer protective effects against these liver conditions [21]. Additionally, the lacto-ovo-vegetarian diet (LOV-D) and the Dietary Approaches to Stop Hypertension (DASHs) diet provide cardiometabolic benefits, including reductions in blood pressure [18], in patients with MASLD.

In addition, certain plant foods, such as whole-grain products, oranges, tea, coffee, flaxseed, and ginger, serve as rich sources of various bioactive compounds, including polyphenols, phytosterols, flavonoids, soluble fiber, and unsaturated fatty acids, whose hepatoprotective properties have been associated with anti-inflammatory and antioxidant effects, as well as a reduction in de novo lipogenesis [14] and improved gut microbiota function [22]. Combining polyphenol-rich plant foods [23] such as walnuts, green tea, and *Wolffia globosa* with the MED [24] (green MED) may offer additional advantages for liver health and MASLD management, allowing for a significantly greater reduction in hepatic steatosis compared to the traditional MED.

This review, by building upon the findings of a previous literature review [18], which suggested that the MED and DASHs may be beneficial for MASLD patients with metabolic comorbidities, aims to explore the specific contribution of individual plant-based foods within these models. The main objective is to identify which food groups or components may exert the most significant effects on mitigating the metabolic complications associated with MASLD, such as BMI, insulin and glycemic control, and liver outcomes. Secondly, the targeted analysis allowing the identification of plant-based foods with the most prominent clinical effects on metabolic improvements will help to enhance the therapeutic utility of plant-based interventions by supporting the development of individualized dietary recommendations for future clinical studies and guidelines dedicated to MASLD prevention and treatment.

## 2. Materials and Methods

This systematic review followed the PRISMA (Preferred Reporting Items for Systematic Reviews and Meta-Analyses) recommendations [25], and the protocol for this study was registered in the PROSPERO system (http://www.crd.york.ac.uk/PROSPERO (accessed on 30 June 2025)) with registration number CRD420251056195 (available from https://www.crd.york.ac.uk/PROSPERO/view/CRD420251056195 (accessed on 30 June 2025)). The key aims of this review, structured based on the patients, intervention, comparison, and outcomes (PICOS) framework, focus on the impact of plant-based single foods and/or food groups on metabolic outcomes in MASLD/NAFLD patients, including those who present with MASLD/NAFLD alone and with obesity, metabolic syndrome, and T2DM.

### 2.1. Search Strategy

A systematic electronic search with predefined criteria was conducted in nine databases, including PubMed, Scopus, Google Scholar, the Cochrane Central Register of Controlled Trials (CENTRAL), Clinical Trials (CT.gov), TRIPP, Web of Science, EMBASE, and EBSCO, to identify randomized controlled trials (RCTs) published between January 2020 and May 2025.

The search criteria were agreed between three researchers, who independently conducted a systematic search with the following terms: “NAFLD”, “Non-alcoholic Fatty Liver Disease”, “MASLD”, “Metabolic Dysfunction-Associated Steatotic Liver Disease”, “obesity”, “type 2 diabetes”, “diabetes mellitus”, and “metabolic syndrome”. These terms were combined with one plant-based product name, such as “Leafy greens”, “Cruciferous vegetables”, “Broccoli”, “Cauliflower”, “Cabbage”, “Tomatoes”, “Sweet potatoes”, “Garlic”, “Jerusalem artichoke”, “Citrus fruits”, “Sweet orange”, “Blueberries”, “Strawberries”, “Raspberries”, “Apples”, “Bananas”, “Grapes”, “Whole grains”, “Edible grain”, “Bread”, “Oats”, “Barley”, “Quinoa”, “Rice”, “Flour”, “Beans”, “Lentils”, “Chickpeas”, “Soybeans”, “Tofu”, “Pea proteins”, “Natto”, “Miso”, “Almonds”, “Walnuts”, “Pistachios”, “Peanuts”, “Chia seeds”, “Flaxseeds”, “Sunflower seeds”, “Hempseed”, “Olive oil”, “Coconut oil”, “Corn oil”, “Rapeseed oil”, “Plant oils”, “Hempseed oil”, “Coffee”, “Tea”, “Green tea”, “Juice”, “Honey”, “Fermented foods”, “Spirulina”, “Chlorella”, “Wakame”, “Curcuma longa”, “Fruit-rich diet”, and “Plant-based”, within the study title and abstract, with the exception of Google Scholar, in which only the title was searched.

The conducted searches were limited to studies in the English language, covering the period between January 2020 and May 2025 (inclusive).

### 2.2. Study Selection

The selection process of RCTs began with three researchers independently screening titles and abstracts, followed by the identification of potentially eligible papers for comprehensive full-text review. Data relevant to pre-established exclusion/inclusion criteria based on the MASLD diagnostic [26] were then retrieved for further analysis.

### 2.3. Selection Criteria

This study exclusively reviewed RCTs that involved a dietary intervention with plant-based single foods and/or food groups and/or powdered forms of these products and included adults aged 18–65 years who had a clinical diagnosis of MASLD/NAFLD using valid methods such as biopsy or ultrasonography, (1) alone or/and with other metabolic conditions, including (2) obesity, (3) metabolic syndrome, and (4) T2DM or insulin resistance.

Manuscripts were excluded from the analysis if they (1) examined supplements and/or medications and/or animal-based products; (2) examined an intervention with model dietary patterns; (3) were focused on patients younger than 18 years or older than 65 years or included pregnant women; (4) were published later than May 2025 and/or earlier than January 2020; and (5) were case–control studies or had an observational design. Moreover, studies combining (6) dietary interventions with lifestyle interventions—resulting in contamination of dietary effects with lifestyle changes such as behavioral interventions, cognitive behavioral therapy, psycho-educational programs, self-management skills, motivational interviewing, and lifestyle changes (physical activity, sleep, stress management, diet quality, and habits)—were also eliminated from the analysis.

### 2.4. Data Extraction

The extracted information from the relevant manuscripts was gathered in an Excel file and included the DOI, title, first author’s family name, publication year, study design, geographical region where the intervention took place, sample size (independently for each intervention and placebo/control), participant characteristics, such as acquired condition and comorbidity, mean age, mean BMI and body weight (BW), gender, and dietary intervention (single foods and/or food groups, intervention duration in weeks, type of control/comparison), as well as baseline and post-intervention measures of metabolic outcomes, including the following:

(1) Anthropometric measures: These include age, body weight (BW), body mass index (BMI), waist circumference (WC), body fat percentage (BF%), and blood pressure (diastolic (DBP) and systolic (SBP)).
(2) Glucose metabolism outcomes: These include fasting glucose, fasting insulin, the Homeostatic Model Assessment of Insulin Resistance (HOMA-IR), and glycated hemoglobin (HbA1c).
(3) Lipid profile: This includes triglycerides (TRGs), total cholesterol (TC), Low-Density Lipoprotein Cholesterol (LDL-C), and High-Density Lipoprotein Cholesterol (HDL-C).
(4) Inflammatory markers: These include High-Sensitivity C-Reactive Protein (hs-CRP) and Lipopolysaccharide (LPS).
(5) Liver function outcomes: These include hepatic enzymes, including aspartate aminotransferase (AST), alanine aminotransferase (ALT), alkaline phosphatase (ALP).
(6) Hepatic steatosis measures: These include the Controlled Attenuation Parameter (CAP), the hepatic inflammation index (HIS) and Fatty Liver Index (FLI), the grade of fatty liver (FL), and liver fibrosis determined by Fibroscan.

The identified interventions with plant-based foods or their extracts are described in the Results Section, and they are detailed in Table 1.

### 2.5. Data Synthesis and Analysis

This study followed the Preferred Reporting Items for Systematic Reviews and Meta-Analyses (PRISMA) recommendations. The extracted information was compiled into summary tables and grouped by categories of metabolic outcomes, including anthropometric measures, glucose and lipid metabolism, and inflammatory outcomes, as well as effects on hepatic function. Comparisons were made both within groups (pre- versus post-intervention) and between intervention and control/placebo groups. A significance threshold of *p* < 0.05 was applied when evaluating changes in outcomes. The results were then interpreted in relation to prior research, with attention to study limitations and the relevance of the findings for clinical application and future investigations.

### 2.6. Risk of Bias Assessment

In this review, the risk of systematic bias was minimized through a comprehensive and systematic literature search conducted across nine databases, including PubMed, Scopus, Google Scholar, the Cochrane Central Register of Controlled Trials (CENTRAL), ClinicalTrials.gov, TRIPP, Web of Science, EMBASE, and EBSCO. The search was performed independently by three authors using predefined inclusion and exclusion criteria, ensuring that all relevant studies were considered and minimizing the possibility of selective reporting. These criteria were established prior to the search to guarantee the inclusion of studies meeting predetermined quality standards. To maintain consistency, only RCTs were included.

To further reduce the risk of systematic bias, a comprehensive search strategy was applied across the nine electronic databases mentioned above. In addition, the search was conducted independently by three researchers, who assessed the identified manuscripts based on the predefined eligibility criteria, ensuring methodological rigor and minimizing selective inclusion.

All studies relevant to the review were critically appraised by the three reviewers before data extraction into tables. A standardized extraction template was used to reduce variability and subjective interpretation. Analyses incorporated all predefined metabolic outcomes to avoid selective reporting. Methodological features such as the study design, sample size, participant characteristics, duration of intervention, type of dietary exposure, and measured outcomes were systematically evaluated to ensure comparability and reliability across studies.

Finally, each RCT was further categorized by its statistical approach, including intention-to-treat (ITT), per-protocol (PP), or as-treated (AT), as described in Table 2.

## 3. Results

**Figure 1:**
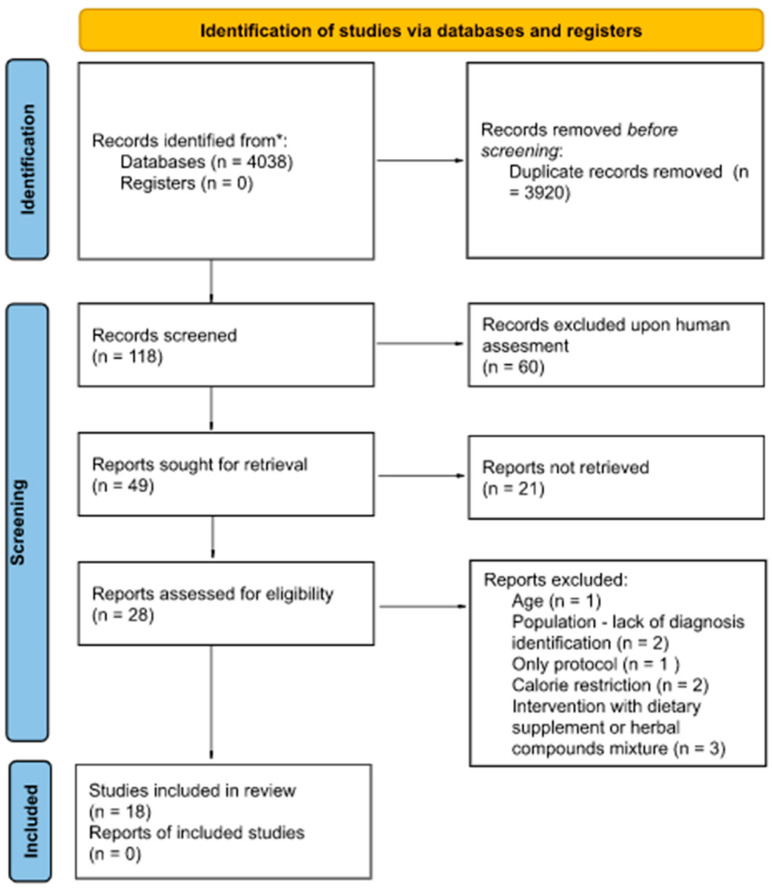
Flowchart with overview of article identification. * in this systematic review the completed clinical trials were included only if they have the protocol and results published and available in the selected databases.

The systematic search through all databases initially identified 4038 manuscripts of interest; however, when duplicates were removed, 49 articles were left for assessment of eligibility, from which 18 studies were selected for this review. The flowchart of this systematic review was obtained with the online tool PRISMA 2020 [25], and it is presented in Figure 1.

### 3.1. Study Characteristics

Characteristics of plant-based foods and their extracts of the 18 reviewed RCT studies are presented in Table 1 and Table 2. The majority of interventions with plant-based products included fruits [27] such as oranges [28], whole-grain products (WGPs) [29,30], oils made of flaxseed [35] or rapeseed plant/canola [38], vegetables (e.g., beetroot, ginger, garlic) [31,40,41,42,43], algae (spirulina [39]), and seeds, including flaxseed varieties [36,37], as well as powdered versions of common beverages, green coffee [32,33] and sour tea [34] (Table 1).

A total number of 1112 participants were included in this systematic review. The analyzed studies primarily focused on patients diagnosed solely with MASLD (15 studies), although some also included patients with comorbidities such as obesity (1 study), metabolic syndrome (1 study), or T2DM and metabolic syndrome (1 study) (Table 2).

The intervention periods ranged from 4 to 24 weeks, with 12-week protocols being the most common among the trials. Based on the inclusion criteria, all studies were RCTs published within the last five years (2020–2025), and the majority of them were carried out in Iran (14 studies), with some conducted in Italy, Poland, Iraq, and China (Table 2).

The mean age of the participants ranged from 35.44 ± 10.85 to 51.8 ± 10.3, and the total sample size in the reviewed articles ranged from 40 to 180 participants (Table 2).

Among the RCTs, the interventions with plant-based foods and their extracts were compared with an equivalent placebo (11 studies), whereas a control group, which was advised to follow their regular dietary habits, was less common (6 studies). There was only one study [30] which used a baseline for comparison with the data recorded at the end of the study (Table 2).

The majority of these RCTs used a powdered form of the plant-based food (eight studies), whereas direct introduction of the food into the diet as a whole food (two studies) or its extract was less common. Interestingly, some interventions included incorporation of the food into oils (two studies), sauce (one study), buns (one study), and beverages, including tea and juice (two studies) (Table 2).

### 3.2. Influence of Dietary Interventions with Plant-Based Foods on Metabolic Outcomes in MASLD Patients

The detailed values of the reported metabolic outcomes in the identified RCTs are presented in Tables 3, 5–7 and 9, while the comparisons of changes between the plant-based food and control/placebo groups are demonstrated in Tables 4, 8 and 10. 

#### 3.2.1. Anthropometric Outcomes

The RCTs presenting effects of interventions with plant-based foods on anthropometric outcomes in MASLD patients are presented in Table 3 and Table 4.

Among the reviewed RCTs, the interventions with plant-based foods, with the exception of the FRD, demonstrated beneficial effects on anthropometric outcomes (Table 3).

The comparison analysis (Table 4) demonstrated that interventions with GCE [32,33], sour tea [34], golden flaxseed [37], and rapeseed oil [38] significantly reduced BW and BMI (*p* < 0.05 for all), whereas including whole oranges [28], WGPs [29], flaxseed oil [35] and powder [36], spirulina sauce [39], and ginger powder [40] had no significant effects compared to the control group (Table 4). Interestingly, mixed effects on BW were reported following supplementation with garlic powder [41,42,43], where only in one study BW was reduced (*p* = 0.001), while in others, it had no significant impact (Table 3 and Table 4).

WC was significantly reduced in studies with BJ [31], sour tea [34], and garlic powder [41,42,43], whereas BF significantly decreased after supplementation with golden flaxseed [37] (*p* < 0.05) and garlic powder [41,42,43] (*p* < 0.001 for all), compared to the control group. However, comparison of these measures in the before–after treatment group revealed further advantages of flaxseed powder consumption [36] for WC. Contrastingly, interventions with whole oranges [28] and HFBs [30] had no significant effect on BF percentage in the reviewed studies (Table 4).

Additional benefits associated with blood pressure management were reported in studies with sour tea [34] and garlic powder [41,42,43], in which both DBP and SBP significantly decreased (*p* < 0.05 for sour tea; *p* < 0.001 for garlic powder), in contrast to spirulina sauce [39], which had no significant effects on these outcomes (Table 4).

Only the FRD [27] demonstrated significant negative effects on anthropometric measures, resulting in increases in BW, BMI, and WC (*p* < 0.001 for all) (Table 3 and Table 4).

#### 3.2.2. Glucose and Lipid Metabolism and Inflammatory Outcomes

The RCT studies reporting impacts of interventions with plant-based foods on glucose, lipid, and inflammatory outcomes in MASLD patients are presented in Table 5, Table 6 and Table 7, and the clinical significance of changes between the intervention and control/placebo groups is reported in Table 8.

Among the reviewed RCTs, the prescription of plant-based foods demonstrated various clinically significant effects on glycemic, lipid, and inflammatory outcomes when compared before and after intervention (Table 5, Table 6 and Table 7).

The analysis of the identified RCTs showed that plant-based foods influenced glycemic management when compared before and after intervention (Table 5). Similar mixed effects were noted for lipid profiles (Table 6) and a few inflammatory indices (Table 7).

For glycemic outcomes, interventions with WGPs [29], rapeseed oil [38], and garlic powder [41,42,43] significantly reduced glucose, insulin, and HOMA-IR indices (*p* < 0.05 for all), whereas flaxseed oil [35] decreased only glucose levels (*p* = 0.016). In contrast, intake of HFBs [30] and golden flaxseed [37] had no significant effects on either glucose or insulin levels compared to the control/placebo (Table 8). Inconsistent effects on glycemic control were reported in several studies, including interventions with whole oranges [28], GCE [32,33], flaxseed powder [36], spirulina sauce [39], and ginger powder [40]. In addition, there were no significant effects on any glycemic indices after intake of HFBs [30] and golden flaxseed powder [37] (Table 8). These results were consistent in a pre–post design comparison (Table 5).

For lipid outcomes, the comparison between interventions demonstrated significant improvements in markers of the lipid profile following intervention with BJ [31] and garlic powder [41,42,43], characterized by reductions in triglycerides, TC, and LDL-C, accompanied by an increase in HDL-C levels. Inconsistent effects on lipid profiles were also reported in studies with GCE [32,33], sour tea [34], and golden [37] and flaxseed powder [36], as well as spirulina sauce [39] and ginger powder [41,42,43]. Interventions with foods rich in dietary fiber, such as WGPs [29] and HFBs [30], showed mixed effects on lipid levels, with certain benefits demonstrated by decreases in TC and LDL-C levels (*p* < 0.05 for all). In addition, there were no significant benefits for any lipid profile outcomes following intake of whole oranges [28] and flaxseed oil [35] (Table 8).

A major improvement in inflammatory status, characterized by a reduction in CRP, was observed in the study with ginger powder [40] (*p* = 0.006), with no significant effects following intake of HFBs [30] or flaxseed powder [36] (Table 8). Interestingly, an intervention with an FRD [27] demonstrated a negative impact on glycemic control and the lipid profile, as it significantly increased glucose, insulin, and the HOMA-IR, as well as triglycerides, total cholesterol, and LDL-C levels (*p* < 0.001 for all) (Table 8).

#### 3.2.3. Liver Function Outcomes

The RCT studies reporting effects of interventions with plant-based foods on liver function outcomes in MASLD patients are presented in Table 9 and Table 10.

The analysis of the reviewed RCTs showed that plant-based foods influenced hepatic enzymes and improved certain liver markers of steatosis and fibrosis when compared before and after intervention (Table 9).

The results obtained have shown that certain plant-based interventions can improve hepatic function; however, the effects of specific foods are mixed (Table 10). Intake of BJ [31] and flaxseed oil [35] significantly reduced all hepatic enzymes (*p* < 0.05 for all), while WGPs [29], sour tea [34], and spirulina sauce [39] decreased AST and ALT (*p* < 0.05 for all) compared to the control group (Table 10). Furthermore, differential effects on certain enzymes were reported for flaxseed powder [36], rapeseed oil [38], and spirulina sauce [39] in comparison with the control (Table 10).

Supplementation with garlic powder [41,42,43] demonstrated inconsistent effects on hepatic enzymes, with studies showing significant reductions in AST and ALT (*p* < 0.05 for all) but no impact on ALP. In contrast, interventions with whole oranges [28], HFBs [30], and GCE [32,33] had no effect on any of the enzymes compared to control groups (Table 10), as well as in pre/post design studies (Table 9). Solely intervention with an FRD [27] demonstrated a significant negative impact on hepatic enzymes, leading to increases in AST, ALT, and ALP both before and after intervention, as well as when compared to the control (Table 9 and Table 10).

Furthermore, a few foods demonstrated a positive effect on hepatic steatosis. Intake of whole oranges [28] and HFBs [30] significantly reduced the CAP compared to the control (Table 10). Interestingly, addition of flaxseed powder to the diet had a significant effect only when assessed in a pre/post-intervention comparison [36] (Table 9) and not when compared to the control (Table 10). Intake of BJ [31], in contrast to ginger powder supplementation [40], significantly reduced hepatic inflammation, as assessed by the FLI, compared to the control (Table 10).

Additionally, interventions with ginger powder demonstrated anti-inflammatory effects in pre/post-intervention comparison (Table 9), as well as when compared to the control (Table 10) [40]. The grade of fatty liver decreased following intervention with WGPs [29] and rapeseed oil [38], with no significant changes after supplementation with GCE [32,33] compared to the control (Table 10).

## 4. Discussion

Adherence to plant-based diets, particularly the MED, has been associated with improved insulin sensitivity, reduced liver fat, and lower hepatic enzyme levels, likely due to their high content of fiber, monounsaturated fats, and polyphenols [19,45]. However, not all plant-derived foods confer benefits, as ultra-processed plant products, fruit juices, or starchy vegetables may increase disease risk [44,46]. Thus, both the quality and degree of processing of plant-based foods are critical for MASLD management. The novelty of this systematic review lies in synthesizing evidence from 18 recent RCTs investigating plant-based foods, highlighting the emerging potential of specific products to improve anthropometric, metabolic, inflammatory, and hepatic outcomes in patients with MASLD and comorbidities such as obesity, metabolic syndrome, or T2DM.

In the present study, dietary supplementation with garlic (400 mg/day) demonstrated significant benefits, including reductions in liver enzymes, such as AST and ALT, and hepatic steatosis, alongside improvements in anthropometric measures, glucose metabolism, the lipid profile, and blood pressure, positioning it as one of the most effective plant-based interventions for MASLD [41,42,43]. Flaxseed exhibited similarly broad metabolic effects, particularly in the context of MASLD-related disturbances [35,36,37]. Both garlic and flaxseed demonstrated significant hepatoprotective effects, with multiple clinical trials and meta-analyses confirming reductions in ALT and AST levels as well as hepatic fat content, assessed via histology or the CAP [36,47,48]. Bioactive compounds in garlic reduce steatosis and hepatic inflammation through antioxidant and immunomodulatory mechanisms [3,49], while flaxseed, rich in α-linolenic acid (ALA) and phytosterols, improves liver outcomes by inhibiting lipogenesis and enhancing β-oxidation [49]. Beyond liver-specific effects, both garlic and flaxseed were associated with improvements in anthropometric measures, including body weight, BMI, and waist circumference [50,51], as well as in glucose and lipid metabolism, including reductions in fasting glucose, insulin, the HOMA-IR, total cholesterol, LDL-C, and triglycerides [52].

Less pronounced effects on metabolic and anthropometric parameters were observed with interventions using WGPs [29] or BJ [31]. Nonetheless, these interventions positively impacted liver outcomes, including hepatic enzymes, the CAP, the FLI, and the degree of steatosis, along with improvements in the lipid profile and glucose metabolism. These effects are consistent with previous evidence indicating that WGPs and BJ improve liver function in MASLD, primarily through reductions in hepatic fat accumulation and serum liver enzymes (ALT, AST), due to their high content of betaine, polyphenols, dietary fiber, and nitrates [3,53]. Dietary fiber and betaine support hepatic lipid metabolism and one-carbon pathways, while WGPs additionally contribute to reductions in hepatic steatosis and waist circumference [29]. Evidence also indicates that whole-grain intake improves lipid profiles by reducing total cholesterol, LDL-C, and triglycerides, increasing HDL-C, and enhancing insulin sensitivity and glycemic control [11,54].

Interventions with sour tea [34], rapeseed oil [38], and spirulina [39] also had positive effects on liver function, reducing liver enzyme levels (e.g., ALT, AST), with rapeseed oil additionally reducing the degree of fatty liver. These plant products, alongside GCE, significantly improved anthropometric outcomes, including BW, BMI, and WC, and metabolic parameters, including fasting glucose, insulin, the HOMA-IR, triglycerides, and total cholesterol, particularly in individuals receiving GCE [32,33] or rapeseed oil [38]. Similar reductions in the HOMA-IR, total cholesterol, LDL-C, and triglycerides were observed for spirulina [39] and ginger [40]. Notably, sour tea was the only intervention that significantly reduced both systolic and diastolic blood pressure [34], consistent with previous dose-dependent findings in individuals with elevated blood pressure [55].

In contrast to the above studies, only the intervention with an FRD [27] demonstrated adverse effects on anthropometric parameters, such as BW, BMI, WC, glucose metabolism, and the lipid profile. The FRD led to significant increases in hepatic enzyme levels, including ALT, AST, and ALP. These opposing effects, compared to other fruit-based interventions, such as daily intake of 400 g of whole oranges [28], underscore the importance of intervention duration. While consumption of oranges for four weeks decreased the CAP score, an FRD over 24 weeks negatively affected hepatic metabolism and other metabolic outcomes. These findings suggest a potential hepatotoxic effect of excessive fruit consumption in MASLD, possibly mediated by high fructose intake and/or caloric overload. In these individuals, excessive fructose has been linked to increased triglyceride synthesis and hepatic steatosis through stimulation of de novo lipogenesis and upregulation of lipogenic enzymes [56], and it bypasses regulatory steps of glycolysis, being metabolized predominantly in the liver, contributing to hepatic lipid accumulation and insulin resistance [57].

This systematic review has several methodological limitations that should be considered when interpreting the findings. The duration of the included interventions varied considerably (4–24 weeks), which may have influenced the observed effects and does not allow for drawing definitive conclusions regarding the efficacy of specific products, particularly those with short-term interventions. Differences in study length introduce variability that makes it hard to compare the effectiveness of the interventions. There was also heterogeneity in the standardization of dietary interventions, with plant-based foods delivered in different forms (e.g., juice, powder, oil, whole products). In several studies, the bioactive compound content was not precisely quantified, making it difficult to evaluate how changes in dose affected the results. Inadequate or inconsistent control of participants’ background diets may have confounded results, as usual dietary patterns could affect metabolic outcomes. Most trials were conducted in Iran, which may introduce geographical bias due to cultural and dietary differences, limiting the generalizability of the findings. Variability in control conditions, including the use of a control group or placebo or the absence of a control arm, may have increased the risk of bias. Moreover, most trials relied on PP analysis, which, while useful for estimating intervention effects under ideal adherence, may overestimate efficacy and introduce selection bias by excluding non-adherent participants. Finally, the small sample sizes in some studies reduced statistical power and increased the likelihood of false-negative results, while variation in study populations (e.g., MASLD with comorbidities) may further limit generalizability.

Despite these limitations, this work has several advantages, including a rigorous methodology, a comprehensive scope, and recent data with focused clinical relevance. By conducting systematic research, the findings presented in this review are consistent and well-structured, demonstrating the transparency of the work. Furthermore, this study focuses on the most recent RCTs with a range of whole plant foods, which are relevant to the most promising dietary models, the MED and DASHs, in the management of MASLD along with its comorbidities.

Given the significant advantage in respect to the limitations of the presented findings, further research is needed to validate the potential of plant-based foods in the other populations long-term. Consequently, prospective investigations should prioritize the design and execution of long-term, high-quality RCTs that rigorously evaluate the efficacy and safety of plant-based foods in the management of MASLD across diverse patient populations. To avoid the homogeneity of cohorts, these studies should include a wider range of age groups, ethnicities, and geographic regions, which allow for generalizability of findings. Dietary interventions should be also structured based on standardized protocols, which define the dosages of foods/bioactive compounds and dosages, while also accounting for dietary and lifestyle factors, as well as comorbidities such as obesity, T2DM, and hypertension. Moreover, the long-term safety, adherence, and sustained impact of plant-based dietary strategies warrant careful examination to support their integration into standardized treatment protocols. Ultimately, building a robust evidence base will facilitate the development of personalized nutrition approaches that optimize metabolic and hepatic outcomes, positioning plant-based foods as a valuable adjunct in comprehensive MASLD management. That approach will enhance the relevance of applying the findings of this review to the clinical settings. Consequently, medical practitioners in collaborations with dieticians may consider addition of the identified plant-based foods and/or their extracts to the diet and/or as adjunctive treatment of patients with MASLD with comorbid conditions such as obesity, T2DM, and elevated blood pressure. The demonstrated effectiveness of these approaches suggest the value of dietary recommendations into specific cases, especially when combined with standardized care tailored to MASLD management and prevention.

## 5. Conclusions

The main findings of this systematic review indicate that plant-based foods may contribute to improvements in metabolic outcomes in MASLD. In addition, the use of certain products, such as WGPs, BJ, whole oranges, and sour tea, may provide additional benefits in these patients, as demonstrated by improved glycemic control, lipid profiles, hepatic functions, and blood pressure management. In contrast, interventions based on an FRD may negatively influence anthropometric and glucose and lipid metabolism indices and increase hepatic enzyme levels. These observations should be interpreted with caution in light of substantial variability in study duration, intervention standardization, and control conditions. Confirmation of these outcomes requires further high-quality, adequately powered RCTs employing standardized protocols to determine the most effective plant-based dietary strategies for the management of MASLD and associated comorbidities, such as obesity and T2DM.

## Figures and Tables

**Table 1:** Plant-based foods included in the reviewed RCTs, along with their form/examples used in the intervention.

Ref.	Name of Plant-Based Food	Form/Examples	Dose	Duration	Additional Comments
Alami et al., 2022 [27]	**A fruit-rich diet (FRD)**	Whole fruit, including colored fruits, dried fruits, and other fruits	At least 4 servings of fruits daily	24 weeks	N/A
Notarnicola et al., 2024 [28]	**Oranges**	Whole “Naveline” oranges	400 g a day	4 weeks	Biological oranges purchased from a BioFarm in Cosenza (Calabria Region, Italy).
Dorosti et al., 2020 [29]	**Whole-grain products (WGPs)**	High-fiber buns (HFBs) baked with fiber obtained from flour (rye) and vital fiber (plantain, psyllium, and apple)	The fiber content was 6.6 ± 0.11 g/roll	8 weeks	Composition of the high-fiber rolls included rye flour type 2000 BIO, vital fiber (20% plantain, 80% psyllium) BIO, apple fiber BIO, ground milk thistle BIO, natural leaven from the fermentation of rye flour type 2000, and yeast. The nutritional content was fat, 2.38 ± 0.11 g/roll; protein, 20.4 ± 0.47 g/roll; and water, 63.7 ± 0.77 g/roll.
Stachowska et al., 2022 [30]		Whole grains including whole wheat, brown rice, oatmeal, whole corn, popcorn, quinoa, barley, buckwheat, bulgur, millet, wild rice, sorghum, amaranth, teff, and triticale	At least half of daily cereal servings obtained from whole-grain cereals	12 weeks	Participants in both groups were asked to eat two to three servings of low-fat dairy products, five servings of fruits and vegetables, and two servings of lean meat, poultry, or fish on a daily basis, as recommended in the 2012 Dietary Guidelines for Americans.
Fateh et al., 2023 [31]	**Beetroot**	Concentrated beetroot juice (BJ)	250 mL of concentrated BJ daily	12 weeks	A 100 mL serving of beetroot juice comprises 95 Kcal energy, 22.6 g carbohydrates, 0.70 g proteins, 0.16 g total lipids, 0.91 g total dietary fiber, and 12 g total sugars. Vitamin C and total flavonoids are within a range of 1.73–7.85 g, 10.75–20.36 mg, and 2.02–2.36 mg (per 100 g).
Hosseinabadi et al., 2020 [32]	**Green coffee**	Green coffee extract (GCE)	A GCE capsule (200 mg) was equal to 1200 mg green coffee bean and 100 mg of CGA obtained from extract	8 weeks	The hydroalcoholic extract of green coffee beans contained 50% CGA and low levels of caffeine (2%).
Hosseinabadi et al., 2020 [33]					
Izadi et al., 2021 [34]	**Sour tea**	Sour tea was made with *Hibiscus sabdariffa* L plant which was obtained from a local market	Sour tea in the form of a 450 mg capsule containing at least 250 mg of anthocyanin	8 weeks	Sour tea was rich in antioxidants (% of weight), primarily anthocyanins (25.46), and anthocyanidins (11.62). It also contained quercetin (7.62), cyanidin (4.78), and unique compounds like hibiscin (4.14), gossypicyanin (3.72), sabdaritrin (3.05), and hibiscitrin (0.98), all contributing to its health-promoting properties. High cellulose content (40.89) reflects its natural fiber composition. The *Hibiscus sabdariffa* L plant used to make tea was obtained from a local market.
Namdar et al., 2024 [35]	**Flaxseed**	Flaxseed oil	Two capsules containing flaxseed oil with total dose prescribed of 1 g two times a day	8 weeks	N/A [35].
Khodadadi et al., 2024 [36]		Flaxseed powder	A portion of 30 g of flaxseed powder per day	12 weeks	
Tian et al., 2025 [37]		Golden flaxseed powder	A serving of 30 g flaxseed powder daily before meals	12 weeks	The flaxseed was golden flaxseed purchased from Canmar Foods Ltd. (Regina, SK, Canada) and contained the following per 100 g: protein—20.0 g; fat—48.7 g (saturated fatty acid—3.3 g; polyunsaturated fatty acid—40.0 g [omega-3 polyunsaturated fatty acid—33.3 g; omega-6 polyunsaturated fatty acid—6.7 g]; monounsaturated fatty acid—5.3 g; trans fatty acid—0 g); carbohydrate—20.0 g (dietary fiber—20.0 g; sugar—0 g); cholesterol—0 g; sodium—66.7 mg; lignan—1.6 g.
Maleki Sedgi et al., 2024 [38]	**Vegetable oil–rapeseed oil**	Rapeseed oil	Three to eight servings of rapeseed oil daily, as part of diet	12 weeks	Regular consumers of ghee consuming from three to eight servings of ghee daily were asked to replace the ghee with rapeseed oil in the same amount.
Mazloomi et al., 2022 [39]	**Spirulina**	Spirulina sauce	One sachet (20 mg) of sauce containing 2 g spirulina per day	8 weeks	Spirulina sauce including spirulina (10%), oil, lemon juice, vinegar, salt, gum, spices, and water.
Rafie et al., 2020 [40]	**Ginger**	Ginger rhizome powder	Three capsules of 500 mg ginger in a powdered form	12 weeks	Ginger powder supplement used in this study is a ready-made product.
Sangouni et al., 2020 [41]	**Garlic**	Garlic powder	Four tablets of powdered garlic daily	15 weeks	Each enteric-coated tablet contained 400 mg garlic powder including 1.5 mg Allicin.
Sangouni et al., 2020 [42]					
Soleimani et al., [43]				12 weeks	

N/A, Not Available.

**Table 2:** The main characteristics of the RCTs analyzed in the systematic review.

Ref.	Country	Population	Number of Participants at the Baseline	Age (Mean ± SD) at the Baseline	Dietary Intervention with Plant-Based Food or Its Extract				Type of Analysis
					Intervention	Duration	Intervention Group	Control/Placebo	ITT, PP, or AT
Alami et al., 2022 [27]	Iran	MASLD	TOTAL: 80; I: 40; C: 40	I: 47.39 ± 10.29; C: 45.11 ± 9.28	FRD	24 weeks	FRD group received at least 4 servings of fruits daily	CONTROL: less than 2 servings daily	PP
Notarnicola et al., 2024 [28]	Italy	MASLD	TOTAL: 62; I: 31; C: 31	I: 51.8 ± 10.3; C: 50.1 ± 9.8	Whole oranges	4 weeks	400 g of whole Navelina variety oranges per day	CONTROL: 400 g of non-citrus fruits daily	ITT
Dorosti et al., 2020 [29]	Iran	MASLD	TOTAL: 112; I: 56; C: 56	I: 43.1 ± 8.9; C: 42.4 ± 8.6	WGPs	12 weeks	At least half of daily cereal servings must be from whole-grain cereals	CONTROL: at least half of daily cereal servings must be from usual grain cereals	PP
Stachowska et al., 2022 [30]	Poland	MASLD	TREATMENT 1—TOTAL: 40 (INTERVENTION ONLY)	MEDIAN: 51.1 (29–68)	HFBs	8 weeks	Replace normal bread in the diet with HFBs divided between two meals a day (2 rolls every day)	NO CONTROL—DATA COMPARED WITH BASELINE	PP
Fateh et al., 2023 [31]	Iraq	MASLD	TOTAL: 180; I: 45; C: 45	I: 44.91 ± 15.24; C: 44.04 ± 13.2	BJ	12 weeks	A 250 mL serving of concentrated BJ given in the morning 30 min before breakfast daily	PLACEBO: A 250 mL glass of water containing red carmoisine food color and a small quantity of a sweetener daily	PP
Hosseinabadi et al., 2020 [32]	Iran	MASLD	TOTAL: 48; I: 24; C: 24	I: 41.14 ± 7.87; C: 41.13 ± 8.47	GCE	12 weeks	A daily dose of 400 mg GCE (2 × 300 mg; *n* = 24)	PLACEBO: placebo capsule similar to GCE tablet in terms of dosage, color, and size containing 200 mg starch	PP
Hosseinabadi et al., 2020 [33]									PP
Izadi et al., 2021 [34]	Iran	MASLD	TOTAL: 70; I: 35; C: 35	I: 43.3 ± 10.2; C: 42.8 ± 10.6	Sour tea	8 weeks	One capsule of sour tea powder (450 mg capsule containing at least 250 mg of anthocyanin) daily	PLACEBO: one placebo capsule (pure microcrystalline cellulose)	PP
Namdar et al., 2024 [35]	Iran	MASLD	TOTAL: 60; I: 30; C: 30	I: 42.23 ± 9.97; C: 38.07 ± 10.40	Flaxseed oil	8 weeks	The dose of capsules containing flaxseed oil was prescribed as 1 g two times a day	PLACEBO: one capsule two times a day	PP
Khodadadi et al., 2024 [36]	Iran	MASLD	TOTAL: 50 I: 25; C: 25	I: 45.07 ± 11.01; C: 45.55 ± 11.59	Flaxseed powder	12 weeks	A portion of 30 g of flaxseed powder per day	CONTROL: received dietary modification recommendations	ITT
Tian et al., 2025 [37]	China	MASLD with obesity	TOTAL: 54; I: 27; C: 27	I: 35.44 ± 10.85; C: 36.32 ± 10.00	Golden flaxseed powder	12 weeks	A serving of 30 g flaxseed powder daily before lunch or dinner along with health education	CONTROL: only health education	PP
Maleki Sedgi et al., 2024 [38]	Iran	MASLD	TOTAL: 60; I: 30; C: 30	Mean total age 42 (SD 9.6) years	Rapeseed oil	12 weeks	Substitute ghee with rapeseed oil in the same amount with a healthy diet	CONTROL: continued the consumption of ghee and was instructed to adhere to a healthy diet	PP
Mazloomi et al., 2022 [39]	Iran	MASLD	TOTAL: 46; I: 23; C: 23	I: 38.87 ± 14.61; C: 35.78 ± 11.14	Spirulina sauce	8 weeks	Spirulina sauce group consumed one sachet (20 mg) of sauce containing 2 g spirulina per day	PLACEBO: one sachet (20 mg) of placebo sauce per day; the placebo sauce was similar in terms of fat, carbohydrate, salt, flavorings, and packaging to the spirulina sauce; to normalize the sensory properties, the color of the sauce, natural dark green chlorophyll was used	ITT
Rafie et al., 2020 [40]	Iran	MASLD	TOTAL: 50; I: 25; C: 25	I: 50.04 ± 10.26; C: 47.95 ± 9.24	Ginger powder	12 weeks	Three capsules containing 500 mg of ginger powder daily	PLACEBO: 3 capsules daily, each containing 500 mg of wheat flour	PP
Soleimani et al., 2020 [43]	Iran	MASLD with T2DM (26 patients) and MS (50 patients)	TOTAL: 110; I: 55; C: 55	I:45.6 ± 11.3; C:42.9 ± 12.21	Garlic powder	15 weeks	Four tablets of powdered garlic daily (each tablet contained 400 mg garlic powder)	PLACEBO: four tablets of placebo containing 400 mg starch	PP
Sangouni et al., 2020 [42]	Iran	MASLD with MS (51 patients)	TOTAL: 110; I: 55; C: 55	I: 46.4 ± 11.3; C: 44.1 ± 11.8	Garlic powder	15 weeks	Four tablets of powdered garlic daily (each tablet contained 400 mg garlic powder)	PLACEBO: placebo tablets in the form of enteric-coated tablets containing 400 mg microcrystalline cellulose	PP
Sangouni et al., 2020 [41]	Iran	MASLD	TOTAL: 90; I: 45; C: 45	I: 45.2 ± 12.4; C:44.2 ± 11.1	Garlic powder	12 weeks	Four tablets of powdered garlic daily (each tablet contained 400 mg garlic powder)	PLACEBO: four tablets of placebo containing 400 mg starch	PP

**Table 3:** Anthropometric outcomes after intervention with plant-based foods in RCTs conducted in MASLD patients.

Dietary Intervention with Plant-Based Food		Anthropometric Outcomes After Intervention with Plant-Based Foods																	
		BW			BMI			WC			BF			DBP			SBP		
		B	PI	*p*	B	PI	*p*	B	PI	*p*	B	PI	*p*	B	PI	*p*	B	PI	*p*
Ref.																			
Alami et al., 2022 [27]	**FRD**	79.4 ± 9.9	86.4 ± 9.5	*p* < 0.001	28.37 ± 2.09	31.40 ± 2.61	*p* < 0.001	109.7 ± 11.3	113.5 ± 10.7	*p* < 0.001	N/A	N/A	N/A	N/A	N/A	N/A	N/A	N/A	N/A
Notarnicola et al., 2024 [28]	**Whole oranges**	91.98 ± 9.96	91.26 ± 9.53	*p* = 0.26	32.07 ± 4.25	31.95 ± 4.28	*p* = 0.57	108.32 ± 12.72	107.32 ± 12.13	*p* = 0.05	29.59 ± 15.94	28.99 ± 13.36	*p* = 0.14	N/A	N/A	N/A	N/A	N/A	N/A
Dorosti et al., 2020 [29]	**WGPs**	87.7 ± 12.0	84.22 ± 11.8	*p* < 0.001	32.5 ± 4.1	32.0 ± 4.2	*p* < 0.001	N/A	N/A	N/A	N/A	N/A	N/A	N/A	N/A	N/A	N/A	N/A	N/A
Stachowska et al., 2022 [30]	**HFBs**	85.2 (59–113.9)		N/A	28.9 (22.8–35.2)		N/A	N/A	N/A	N/A	27.4 (11.7–43.6)		N/A	N/A	N/A	N/A	N/A	N/A	N/A
Fateh et al., 2023 [31]	**BJ**	N/A	N/A	N/A	29.19 ± 3.94	27.75 ± 3.74	*p* < 0.001	91.34 ± 4.53	89.91 ± 4.77	*p* < 0.001	N/A	N/A	N/A	N/A	N/A	N/A	N/A	N/A	N/A
Hosseinabadi et al., 2020 [32]	**GCE**	85.95 ± 11.76	84.30 ± 12.17	*p* < 0.001	30.14 ± 2.60	29.54 ± 2.59	*p* < 0.001	N/A	N/A	N/A	N/A	N/A	N/A	N/A	N/A	N/A	N/A	N/A	N/A
Hosseinabadi et al., 2020 [33]		85.95 ± 11.76	84.30 ± 12.17	*p* < 0.001	30.14 ± 2.60	29.54 ± 2.59	*p* < 0.001	N/A	N/A	N/A	N/A	N/A	N/A	N/A	N/A	N/A	N/A	N/A	N/A
Izadi et al., 2021 [34]	**Sour tea**	87.72 ± 15.7	86.38 ± 16.1	*p* < 0.001	31.65 ± 4.8	31.24 ± 4.8	*p* = 0.001	110.28 ± 13.08	109.3 ± 12.4	*p* = 0.012	N/A	N/A	N/A	8.03 ± 1.8	7.4 ± 1.01	*p* = 0.02	12.75 ± 1.7	11.1 ± 0.60	*p* < 0.001
Namdar et al., 2024 [35]	**Flaxseed oil**	85.93 ± 13.17	84.08 ± 9.50	*p* = 0.089	29.14 ± 3.50	28.59 ± 2.97	*p* = 0.094	98.23 ± 8.18	97.03 ± 6.70	*p* = 0.372	N/A	N/A	N/A	N/A	N/A	N/A	N/A	N/A	N/A
Khodadadi et al., 2024 [36]	**Flaxseed powder**	84.66 ± 15.34	78.06 ± 12.74	*p* < 0.001	30.37 ± 4.41	28.05 ± 3.89	*p* < 0.001	100.08 ± 8.63	90.58 ± 19.32	*p* = 0.028	N/A	N/A	N/A	N/A	N/A	N/A	N/A	N/A	N/A
Tian et al., 2025 [37]	**Golden flaxseed powder**	90.5 ± 12.1	88.1 ± 10.6	NS	31.33 (29.50, 34.40)	30.67 (28.72, 32.62)	NS	N/A	N/A	N/A	38.02 ± 5.10	31.62 ± 3.62	*p* < 0.05	N/A	N/A	N/A	N/A	N/A	N/A
Maleki Sedgi et al., 2024 [38]	**Rapeseed oil**	81.1 ± 8.5	76.8 ± 9.1	*p* < 0.001	28.1 ± 1.7	26.6 ± 1.8	*p* < 0.001	N/A	N/A	N/A	N/A	N/A	N/A	N/A	N/A	N/A	N/A	N/A	N/A
Mazloomi et al., 2022 [39]	**Spirulina sauce**	69.34 ± 10.09	68.54 ± 9.10	*p* = 0.06	24.82 ± 2.87	24.60 ± 2.88	*p* = 0.07	93.78 ± 6.29	93.10 ± 5.11	*p* = 0.26	N/A	N/A	N/A	87.60 ± 8.02	85.05 ± 6.07	*p* = 0.17	130.04 ± 7.05	126.95 ± 7.12	*p* = 0.14
Rafie et al., 2020 [40]	**Ginger powder**	88.61 ± 11.50	86.34 ± 10.85	*p* < 0.001	31.78 ± 3.71	30.96 ± 3.41	*p* < 0.001	105.15 ± 7.26	103.84 ± 7.25	*p* < 0.001	N/A	N/A	N/A	N/A	N/A	N/A	N/A	N/A	N/A
Soleimani et al., [43]	**Garlic powder**	82.6 ± 14.3	80.4 ± 14	*p* = 0.001	30.7 ± 5.2	N/A	N/A	N/A	N/A	N/A	27.7 ± 8.1	N/A	N/A	N/A	N/A	N/A	N/A	N/A	N/A
Sangouni et al., 2020 [42]	**Garlic powder**	I Group: 82.4 ± 14	N/A	30.7 ± 5.3	N/A	N/A	95.2 ± 10.7	N/A	N/A	N/A	N/A	N/A	8.9 ± 1.1	−4 ± 0.84 ^	N/A	13.3 ± 1.2	−6.74 ± 1.25 ^	N/A	
Sangouni et al., 2020 [41]	**Garlic powder**	89.8 ± 11.9	89.2 ± 11.8	N/A	30.2 ± 3.1	30.0 ± 3.1	N/A	105.6 ± 9.8	104.0 ± 0	N/A	N/A	N/A	N/A	N/A	N/A	N/A	N/A	N/A	N/A

Data is presented as Mean ± SD or Median (25th–75th). Baseline (B) and post-intervention (PI) reported as exact values or otherwise marked ^ and displayed as MD. N/A means that there was no data available. BW expressed in kilograms (kg). BMI expressed in kg/m^2^. WC expressed in cm. BF expressed as %. SBP and DBP are expressed as mmHg. *p* value > 0.05 results considered as NS. BW, body weight; BMI, body mass index; WC, waist circumference; BF, body fat; DBP, diastolic blood pressure; SBP, systolic blood pressure; MD, Mean Difference; SD, Standard Deviation; NS, not significant; N/A, Not Available; B, baseline; PI, post-intervention; fruit-rich diet, FRD; whole-grain products, WGPs; beetroot juice, BJ; green coffee extract, GCE.

**Table 4:** Comparisons of changes in anthropometric outcomes after interventions with plant-based foods and control/placebo in RCTs conducted in MASLD patients.

Change in Anthropometric Outcomes Following Interventions with Plant-Based Foods in MASLD						
Compared Plant-Based Foods	BW	BMI	WC	BF	DBP	SBP
FRD [27]	*p* < 0.001 *	*p* < 0.001 *	*p* < 0.001 *	N/A	N/A	N/A
Whole oranges [28]	*p* = 0.43	*p* = 0.17	*p* = 0.91	*p* = 0.97	N/A	N/A
WGPs [29]	*p* = 0.34	*p* = 0.65	*p* = 0.10	N/A	N/A	N/A
HFBs [30]	*p* = 0.35	*p* = 0.057	N/A	*p* = 0.18	N/A	N/A
BJ [31]	N/A	*p* = 0.191	*p* = 0.008	N/A	N/A	N/A
GCE [32,44]	*p* < 0.001	*p* < 0.001	N/A	N/A	N/A	N/A
				N/A	N/A	N/A
Sour tea [34]	*p* < 0.05	*p* < 0.05	*p* < 0.05	N/A	*p* < 0.05	*p* < 0.05
Flaxseed oil [35]	*p* = 0.052	*p* = 0.662	*p* = 0.175	N/A	N/A	N/A
Flaxseed powder [36]	*p* = 0.058	*p* = 0.058	*p* = 0.219	N/A	N/A	N/A
Golden flaxseed powder [37]	*p* < 0.05	*p* < 0.05	N/A	*p* < 0.05	N/A	N/A
Rapeseed oil [38]	*p* < 0.001	*p* < 0.001	N/A	N/A	N/A	N/A
Spirulina sauce [39]	*p* = 0.16	*p* = 0.57	*p* = 0.35	N/A	*p* = 0.06	*p* = 0.68
Ginger powder [40]	*p* = 0.773	*p* = 0.544	*p* = 0.221	N/A	N/A	N/A
Garlic powder [41,42,43]	*p* = 0.010	N/A	N/A	N/A	N/A	N/A
	N/A	N/A	N/A	N/A	*p* < 0.001	*p* < 0.001
	*p* = 0.86	*p* = 0.12	*p* = 0.001	*p* < 0.001	N/A	N/A
	*p* = 0.86	*p* = 0.12	*p* = 0.001	*p* < 0.001	N/A	N/A

Data is presented as a *p* value and color-coded according to the level of significance of the change in reported PI values after interventions with plant-based foods. The *p* values demonstrating a significant increase in the reviewed outcomes are marked with *. N/A means that there was no data available. BW, body weight; BMI, body mass index; WC, waist circumference; BF, body fat; DBP, diastolic blood pressure; SBP, systolic blood pressure; NS, not significant; fruit-rich diet, FRD; whole-grain products, WGPs; beetroot juice, BJ; green coffee extract, GCE.

**Table 5:** Outcomes of glucose metabolism after intervention with plant-based foods in RCTs conducted in MASLD patients.

Dietary Intervention with Plant-Based Food		Glucose Metabolism Outcomes After Intervention with Plant-Based Foods											
		Glucose			Insulin			HOMA-IR			HbA1c		
		B	PI	*p*	B	PI	*p*	B	PI	*p*	B	PI	*p*
Ref.													
Alami et al., 2022 [27]	**FRD**	96.9 ± 9.4	115.5 ± 30.0	*p* < 0.001	14.0 ± 5.7	26.6 ± 15.9	*p* < 0.001	3.32 ± 1.41	7.36 ± 4.37	*p* < 0.001	N/A	N/A	N/A
Notarnicola et al., 2024 [28]	**Whole oranges**	101.00 ± 21.62	99.59 ± 26.83	*p* = 0.72	16.08 ± 8.55	16.42 ± 9.06	*p* = 0.36	4.10 ± 2.50	4.19 ± 2.94	*p* = 0.99	N/A	N/A	N/A
Dorosti et al., 2020 [29]	**WGPs**	88.1 ± 10.2	86.9 ± 8.2	NS	17.0 ± 9.3	14.9 ± 7.9	*p* < 0.05	3.5 ± 0.3	3.2 ± 0.3	*p* < 0.05	N/A	N/A	N/A
Stachowska et al., 2022 [30]	**HFBs**	96.1 (76.3–272.6)		N/A	18.5 (4.3–129)		N/A	N/A	N/A	N/A	N/A	N/A	N/A
Fateh et al., 2023 [31]	**BJ**	N/A	N/A	N/A	N/A	N/A	N/A	N/A	N/A	N/A	N/A	N/A	N/A
Hosseinabadi et al., 2020 [32]	**GCE**	104.65 ± 9.09	92.15 ± 11.40	*p* < 0.001	10.22 ± 4.04	10.41 ± 4.29	*p* = 0.871	2.65 ± 1.09	2.42 ± 1.20	*p* = 0.463	N/A	N/A	N/A
Hosseinabadi et al., 2020 [33]		N/A	N/A	N/A	N/A	N/A	N/A	N/A	N/A	N/A	N/A	N/A	N/A
Izadi et al., 2021 [34]	**Sour tea**	N/A	N/A	N/A	N/A	N/A	N/A	N/A	N/A	N/A	N/A	N/A	N/A
Namdar et al., 2024 [35]	**Flaxseed oil**	98.67 ± 19.35	98.53 ± 22.44	*p* = 0.776	N/A	N/A	N/A	N/A	N/A	N/A	N/A	N/A	N/A
Khodadadi et al., 2024 [36]	**Flaxseed powder**	104.00 ± 8.88	97.87 ± 8.02	*p* < 0.001	10.99 ± 8.87	6.63 ± 5.77	*p* < 0.001	2.80 ± 2.16	1.58 ± 1.32	*p* < 0.001	N/A	N/A	N/A
Tian et al., 2025 [37]	**Golden flaxseed powder**	5.06 ± 0.56	5.10 ± 0.49	NS	113.36 (92.70, 136.60)	106.86 (95.30, 146.40)	NS	N/A	N/A	N/A	N/A	N/A	N/A
Maleki Sedgi et al., 2024 [38]	**Rapeseed oil**	98.7 ± 9.6	89.2 ± 9.3	*p* < 0.001	13.2 ± 6.8	10.1 ± 5.3	*p* = 0.002	3.2 ± 1.7	2.3± 1.4	*p* = 0.001	N/A	N/A	N/A
Mazloomi et al., 2022 [39]	**Spirulina sauce**	91.43 ± 7.71	87.20 ± 7.80	*p* = 0.18	8.30 ± 3.27	7.57 ± 2.36	*p* = 0.29	1.90 ± 0.82	1.63 ± 0.56	*p* = 0.10	N/A	N/A	N/A
Rafie et al., 2020 [40]	**Ginger powder**	107.52 ± 10.64	99.34 ± 12.57	*p* = 0.007	13.38 ± 2.75	12.42 ± 2.53	*p* = 0.017	3.72± 0.76	3.07 ± 0.80	*p* = 0.001	N/A	N/A	N/A
Soleimani et al. [43]	**Garlic powder**	124.2 ± 37	115.8 ± 39.3	*p* = 0.001	N/A	N/A	N/A	N/A	N/A	N/A	6.27 ± 1.5	6.04 ± 1.6	*p* = 0.028
Sangouni et al., 2020 [42]	**Garlic powder**	N/A	N/A	N/A	N/A	N/A	N/A	N/A	N/A	N/A	N/A	N/A	N/A
Sangouni et al., 2020 [41]	**Garlic powder**	90.1 ± 7.8	86.9 ± 8.2	N/A	8.5 ± 2.7	5.6 ± 2.5	N/A	1.88 ± 0.6	1.21 ± 0.5	N/A	N/A	N/A	N/A

Data is presented as Mean ± SD or Median (25–75th). B and PI reported as exact values or otherwise marked and displayed as MD. N/A means that there was no data available. Glucose expressed in mg/dL. Insulin expressed in µU/mL. HbA1c expressed in %. *p* value > 0.05 results considered as NS. HOMA-IR, Homeostatic Model Assessment of Insulin Resistance; HbA1c, hemoglobin A1c; MD, Mean Difference; SD, Standard Deviation; NS, not significant; N/A, Not Available; B, baseline; PI, post-intervention; fruit-rich diet, FRD; whole-grain products, WGPs; beetroot juice, BJ; green coffee extract, GCE.

**Table 6:** Outcomes of lipid metabolism after intervention with plant-based foods in RCTs conducted in MASLD patients.

Dietary Intervention with Plant-Based Food		Lipid Metabolism Outcomes After Intervention with Plant-Based Foods											
		Triglycerides			Cholesterol			LDL-C			HDL-C		
		B	PI	*p*	B	PI	*p*	B	PI	*p*	B	PI	*p*
Alami et al., 2022 [27]	**FRD**	183.2 ± 100.8	248.6 ± 125.0	*p* < 0.001	174.6 ± 35.5	206.1 ± 40.5	*p* < 0.001	99.9 ± 29.4	126.9 ± 32.3	*p* < 0.001	50.4 ± 11.1	41.4 ± 8.9	*p* < 0.001
Notarnicola et al., 2024 [28]	**Whole oranges**	132.10 ± 53.32	123.06 ± 53.55	*p* = 0.72	202.29 ± 40.25	193.39 ± 40.83	*p* = 0.28	132.11 ± 37.64	130.94 ± 35.58	*p* = 0.47	47.10 ± 13.15	47.53 ± 10.74	*p* = 0.72
Dorosti et al., 2020 [29]	**WGPs**	167.9± 13.6	156.7 ± 11.6	NS	192.4 ± 40.9	174.1 ± 37.3	*p* < 0.001	114.4 ± 4.5	101.5 ± 4.2	*p* < 0.05	41.2 ± 7.0	43.0 ± 5.9	*p* < 0.05
Stachowska et al., 2022 [30]	**HFBs**	150.5 (50.9–452)		N/A	178.2 (98–340.2)		N/A	114.4 (47.9–258.3)		N/A	44.9 (25.8–77.5)		N/A
Fateh et al., 2023 [31]	**BJ**	233.5 ± 14.1	217.6 ± 11.6	*p* < 0.001	228.9 ± 6.2	210.5 ± 3.7	*p* < 0.001	140.2 ± 9.2	132.2 ± 5.8	*p* < 0.001	29.8 ± 3.9	36.6 ± 4.2	*p* < 0.001
Hosseinabadi et al., 2020 [32]	**GCE**	N/A	N/A	N/A	N/A	N/A	N/A	N/A	N/A	N/A	N/A	N/A	N/A
Hosseinabadi et al., 2020 [33]		187.04 ± 106.04	159.14 ± 74.96	*p* = 0.10	231.66 ± 43.70	218.33± 38.52	*p* = 0.04	138.25 ± 29.55	127.92 ±30.87	*p* = 0.06	55.04 ± 10.03	58.47 ± 8.71	*p* = 0.08
Izadi et al., 2021 [34]	**Sour tea**	165.9 ± 74.5	146.8 ± 61.2	*p* = 0.008	197.2 ± 45.4	188.6 ± 45.1	*p* = 0.03	122.6 ± 18.9	112.9 ± 20.7	*p* = 0.008	41.6 ± 8.5	43.2 ± 9.4	*p* = 0.130
Namdar et al., 2024 [35]	**Flaxseed oil**	207.27 ± 86.38	188.77 ± 61.54	*p* = 0.371	203.17 ± 41.64	215.83 ± 32.97	*p* = 0.175	120.3 ± 38.94	110.83 ± 29.20	*p* = 0.066	40.96 ± 11.79	45.57 ± 9.06	*p* = 0.003
Khodadadi et al., 2024 [36]	**Flaxseed powder**	206.17 ± 82.01	144.83 ± 59.76	*p* < 0.001	203.42 ± 35.00	171.70 ± 26.49	*p* < 0.001	125.93 ± 27.12	103.28 ± 22.62	*p* < 0.001	36.25 ± 13.82	39.45 ± 12.31	*p* = 0.072
Tian et al., 2025 [37]	**Golden flaxseed powder**	2.11 ± 0.74	1.71 ± 0.63	*p* < 0.05	5.22 ± 0.80	4.75 ± 0.78	*p* < 0.05	3.43 ± 0.76	3.20 ± 0.98	NS	1.01 ± 0.09	1.12 ± 0.17	*p* < 0.05
Maleki Sedgi et al., 2024 [38]	**Rapeseed oil**	N/A	N/A	N/A	184.4 ±50	167.2 ± 40.8	*p* < 0.001	106.6 ± 99.7	99.7 ± 28.4	*p* = 0.008	N/A	N/A	N/A
Mazloomi et al., 2022 [39]	**Spirulina sauce**	165.30 ± 41.20	138.65 ± 41.70	*p* = 0.03	202.48 ± 45	186.75 ± 49.86	*p* = 0.14	126.96 ± 45.18	116.60 ± 41.76	*p* = 0.11	42.43 ± 8.29	46.40 ± 11.64	*p* = 0.02
Rafie et al., 2020 [40]	**Ginger powder**	200.60 ± 48.56	196.43 ± 46.24	*p* = 0.503	220.82 ±45.95	196.13 ±36.23	*p* = 0.006	136.59 ± 45.70	113.56 ± 37.90	*p* = 0.010	43.69 ± 7.43	44.73 ± 6.54	*p* = 0.341
Sangouni et al., 2020 [41]	**Garlic powder**	169.2 ± 67.5	148.8 ± 74.7	*p* = 0.002	184.2 ± 32.5	171.4 ± 31.9	*p* = 0.005	111.5 ± 28.1	99.1 ± 27.5	*p* = 0.002	40.5 ± 8.8	42.7 ± 10.22	*p* = 0.06
Sangouni et al., 2020 [42]	**Garlic powder**	N/A	N/A	N/A	N/A	N/A	N/A	N/A	N/A	N/A	N/A	N/A	N/A
[41]	**Garlic powder**	2.1 ± 0.9	1.7 ± 0.7	N/A	5.3 ± 1.0	5.0 ± 0.9	N/A	3.2 ± 0.7	2.9 ± 0.6	N/A	1.1 ± 0.2	1.2 ± 0.2	N/A

Data is presented as Mean ± SD or Median (25th-75th). B and PI reported as exact values or otherwise marked and displayed as MD. N/A means that there was no data available. Triglycerides, cholesterol, LDL-C, and HDL-C expressed in mg/dL or otherwise marked by * and presented in mmol/L. *p* value > 0.05 results considered as NS. HDL-C, High-Density Lipoprotein Cholesterol; LDL-C, Low-Density Lipoprotein Cholesterol; MD, Mean Difference; SD, Standard Deviation; NS, not significant; N/A, Not Available; B, baseline; PI, post-intervention; fruit-rich diet, FRD; whole-grain products, WGPs; beetroot juice, BJ; green coffee extract, GCE.

**Table 7:** Outcomes of inflammatory metabolism after intervention with plant-based foods in RCTs conducted in MASLD patients.

Dietary Intervention with Plant-Based Food		Inflammatory Outcomes After Intervention with Plant-Based Foods					
		hs-CRP			LPS (pg/mL)		
		B	PI	*p*	B	PI	*p*
Alami et al., 2022 [27]	**FRD**	N/A	N/A	N/A	N/A	N/A	N/A
Notarnicola et al., 2024 [28]	**Whole oranges**	0.34 ± 0.43	0.30 ± 0.41	*p* = 0.58	N/A	N/A	N/A
Dorosti et al., 2020 [29]	**WGPs**	N/A	N/A	N/A	N/A	N/A	N/A
Stachowska et al., 2022 [30]	**HFBs**	N/A	N/A	N/A	153 (0–481)		N/A
Fateh et al., 2023 [31]	**BJ**	N/A	N/A	N/A	N/A	N/A	N/A
Hosseinabadi et al., 2020 [32]	**GCE**	N/A	N/A	N/A	N/A	N/A	N/A
Hosseinabadi et al., 2020 [33]		N/A	N/A	N/A	N/A	N/A	N/A
Izadi et al., 2021 [34]	**Sour tea**	N/A	N/A	N/A	N/A	N/A	N/A
Namdar et al., 2024 [35]	**Flaxseed oil**	N/A	N/A	N/A	N/A	N/A	N/A
Khodadadi et al., 2024 [36]	**Flaxseed powder**	4.70 ± 2.07	3.47 ± 1.46	*p* = 0.012	N/A	N/A	N/A
Tian et al., 2025 [37]	**Golden flaxseed powder**	N/A	N/A	N/A	N/A	N/A	N/A
Maleki Sedgi et al., 2024 [38]	**Rapeseed oil**	N/A	N/A	N/A	N/A	N/A	N/A
Mazloomi et al., 2022 [39]	**Spirulina sauce**	N/A	N/A	N/A	N/A	N/A	N/A
Rafie et al., 2020 [40]	**Ginger powder**	2.40 (1.14, 3.58)	1.82 (0.88, 3.18)	*p* = 0.001	N/A	N/A	N/A
Sangouni et al., 2020 [41]	**Garlic powder**	N/A	N/A	N/A	N/A	N/A	N/A
Sangouni et al., 2020 [42]	**Garlic powder**	N/A	N/A	N/A	N/A	N/A	N/A
Soleimani et al. [43]	**Garlic powder**	N/A	N/A	N/A	N/A	N/A	N/A

Data is presented as Mean ± SD or Median (25th–75th). B and PI reported as exact values or otherwise marked and displayed as MD. N/A shows where there was no data available. hs-CRP expressed in ng/mL. LPS expressed in pg/mL. *p* value > 0.05 results considered as NS. hs-CRP, High-Sensitivity C-Reactive Protein; LPS, Lipopolysaccharide; MD, Mean Difference; SD, Standard Deviation; NS, not significant; N/A, Not Available; B, baseline; PI, post-intervention; fruit-rich diet, FRD; whole-grain products, WGPs; beetroot juice, BJ; green coffee extract, GCE.

**Table 8:** Comparisons of changes in glucose, lipid, and inflammatory outcomes after interventions with plant-based foods and control/placebo in RCTs conducted in MASLD patients.

Change in Glucose and Lipid Metabolism Outcomes Along with Inflammatory Status After Interventions with Plant-Based Foods in MASLD										
Compared Plant-Based Foods	Glucose	Insulin	HOMA-IR	HbA1c	Triglycerides	Cholesterol	LDL-C	HDL-C	hs-CRP	LPS
FRD [27]	*p* < 0.001 *	*p* < 0.001 *	*p* < 0.001 *	N/A	*p* < 0.001 *	*p* < 0.001 *	*p* < 0.001 *	*p* < 0.001	N/A	N/A
Whole oranges [28]	*p* = 0.09	*p* = 0.58	*p* = 0.94	N/A	*p* = 0.66	*p* = 0.07	*p* = 0.69	*p* = 0.92	*p* = 0.79	N/A
WGPs [29]	*p* = 0.020	*p* = 0.015	*p* = 0.016	N/A	*p* = 0.11	*p* = 0.004	*p* = 0.014	*p* = 0.54	N/A	N/A
HFBs [30]	*p* = 0.63	*p* = 0.52	N/A	N/A	*p* = 0.14	*p* = 0.04	*p* = 0.06	*p* = 0.36	N/A	*p* = 1
BJ [31]	N/A	N/A	N/A	N/A	*p* < 0.001	*p* < 0.001	*p* < 0.001	*p* < 0.001	N/A	N/A
GCE [32,44]	*p* = 0.006	*p* = 0.113	*p* = 0.028	N/A	N/A	N/A	N/A	N/A	N/A	N/A
	N/A	N/A	N/A	N/A	*p* = 0.32	*p* = 0.36	*p* = 0.33	*p* = 0.04	N/A	N/A
Sour tea [34]	N/A	N/A	N/A	N/A	*p* = 0.03	*p* = 0.61	*p* = 0.55	*p* = 0.55	N/A	N/A
Flaxseed oil [35]	*p* = 0.016	N/A	N/A	N/A	*p* = 0.947	*p* = 0.420	*p* = 0.520	*p* = 0.80	N/A	N/A
Flaxseed powder [36]	*p* = 0.379	*p* < 0.001	*p* = 0.002	N/A	*p* < 0.001	*p* = 0.028	*p* = 0.552	*p* = 0.638	*p* = 0.598	N/A
Golden flaxseed powder [37]	NS	NS	N/A	N/A	*p* < 0.05	NS	*p* < 0.05	*p* < 0.05	N/A	N/A
Rapeseed oil [38]	*p* < 0.001	*p* < 0.001	*p* < 0.001	N/A	N/A	*p* = 0.006	*p* = 0.07	N/A	N/A	N/A
Spirulina sauce [39]	*p* = 0.55	*p* = 0.08	*p* = 0.047	N/A	*p* = 0.02	*p* = 0.15	*p* = 0.17	*p* = 0.07	N/A	N/A
Ginger powder [40]	*p* = 0.029	*p* = 0.559	*p* = 0.047	N/A	*p* = 0.823	*p* = 0.026	*p* = 0.032	*p* = 0.948	*p* = 0.006	N/A
Garlic powder [41,42,43]	*p* = 0.001	N/A	N/A	*p* = 0.001	*p* = 0.022	*p* = 0.005	*p* = 0.005	*p* = 0.556	N/A	N/A
	N/A	N/A	N/A	N/A	N/A	N/A	N/A	N/A	N/A	N/A
	N/A	N/A	N/A	N/A	*p* < 0.001	*p* = 0.02	*p* = 0.01	*p* < 0.001	N/A	N/A
	*p* = 0.02	*p* = 0.001	*p* < 0.001	N/A	N/A	N/A	N/A	N/A	N/A	N/A

Data is presented as a *p* value and color-coded according to the level of significance of the change in reported PI values after interventions with plant-based foods. The *p* values demonstrating a significant increase in the reviewed outcomes are marked with *. N/A means that there was no data available. HOMA-IR, Homeostatic Model Assessment of Insulin Resistance; HbA1c, hemoglobin A1c; HDL-C, High-Density Lipoprotein Cholesterol; LDL-C, Low-Density Lipoprotein Cholesterol; CRP, High-Sensitivity C-Reactive Protein; LPS, Lipopolysaccharide; NS, not significant; N/A, Not Available; fruit-rich diet, FRD; whole-grain products, WGPs; beetroot juice, BJ; green coffee extract, GCE.

**Table 9:** Liver function after intervention with plant-based foods in RCTs conducted in MASLD patients.

Dietary Intervention with Plant-Based Food		Liver Function Outcomes After Intervention with Plant-Based Foods														
		Hepatic Enzymes									Hepatic Steatosis					Liver Fibrosis
		AST			ALT			ALP			CAP			Hepatic Inflammation (FLI)	Grade of Fatty Liver	
		B	PI	*p*	B	PI	*p*	B	PI	*p*	B	PI	*p*			
Alami et al., 2022 [27]	**FRD**	26.8 ± 11.0	74.5 ± 107.8	*p* < 0.001	38.1 ± 25.3	89.1 ± 92.9	*p* < 0.001	189.4 ± 73.2	273.4 ± 128.5	*p* < 0.001	N/A	N/A	N/A	N/A	N/A	N/A
Notarnicola et al., 2024 [28]	**Whole oranges**	23.42 ± 9.93	24.29 ± 7.29	*p* = 0.05	36.68 ± 23.74	34.93 ± 18.50	*p* = 0.28	67.64 ± 19.99	68.74 ± 19.88	*p* = 0.58	N/A	N/A	N/A	N/A	N/A	N/A
Dorosti et al., 2020 [29]	**WGPs**	27.7 ± 13.6	21.9 ± 6.8	*p* < 0.001	34.6 ± 12.5	24.1 ± 12.2	*p* < 0.001	N/A	N/A	N/A	N/A	N/A	N/A	N/A	B—Normal Liver: 0; Grade 1: 24 (51.1%); Grade 2: 20 (42.6%); Grade 3: 3 (6.4%); PI—Normal Liver: 17 (36.2%); Grade 1: 23 (48.9%); Grade 2: 7 (14.9%); Grade 3: 0 (0%)	N/A
Stachowska et al., 2022 [30]	**HFBs**	24 (13–40)		N/A	35 (11–86)		N/A	N/A	N/A	N/A	277 (224–371)/95% CI: 274.94–310.68		N/A	N/A	N/A	FibroScan: 5.3 (3.6–9.7)/95% CI: 5.02–6.13
Fateh et al., 2023 [31]	**BJ**	61.43 ± 8.85	58.18 ± 6.33	*p* < 0.001	37.63 ± 3.45	36.61 ± 5.87	*p* = 0.320	119.0 ± 8.5	113.6 ± 7.6	*p* < 0.001	N/A	N/A	N/A	FLI: B—79; PI—42; NS	Change in the liver fat content after intervention—PI—reduction 1 Grade: 23; reduction 2 Grades: 10; no change: 12	N/A
Hosseinabadi et al., 2020 [32], Hosseinabadi et al., 2020 [33]	**GCE**	35.71 ± 22.63	32.66 ± 16.74	*p* = 0.48	43.85 ± 25.82	44.52 ± 30.08	*p* = 0.90	N/A	N/A	N/A	N/A	N/A	N/A	N/A	B—Normal Liver: 0; Grade 1: 12; Grade 2: 8; Grade 3: 1; PI—Normal Liver: 1; Grade 1: 12; Grade 2: 8; Grade 3: 0	N/A
Izadi et al., 2021 [34]	**Sour tea**	45.5 ± 13.4	39.8 ± 12.7	*p* = 0.04	35.16 ± 18.5	30.53 ± 13.4	*p* = 0.01	N/A	N/A	N/A	N/A	N/A	N/A	N/A	N/A	N/A
Namdar et al., 2024 [35]	**Flaxseed oil**	54.5 ± 19.87	33.37 ± 8.07	*p* < 0.001	89.13 ± 39.30	48.17 ± 14.11	*p* < 0.001	209 ± 50.26	167.43 ± 38.15	*p* < 0.001	N/A	N/A	N/A	N/A	B—Normal Liver: 0 (0%); Grade 1: 0 (0%); Grade 2: 48 (87.3%); Grade 3: 7 (12.7%); PI—Normal Liver: 8 (14.54%); Grade 1: 31 (56.36%); Grade 2: 16 (9.1%); Grade 3: 0 (0%)	N/A
Khodadadi et al., 2024 [36]	**Flaxseed powder**	28.13 ± 16.94	17.00 ± 7.05	*p* < 0.001	24.67 ± 8.39	19.29 ± 5.83	*p* = 0.003	N/A	N/A	N/A	306.62 ± 32.77	259.62 ± 38.48	*p* < 0.001	N/A	N/A	Fibrosis score—B: 6.01 ± 1.96; PI: 4.75 ± 1.29; *p* < 0.001
Tian et al., 2025 [37]	**Golden flaxseed powder**	23.00 (21.50, 28.00)	18.00 (16.00, 23.00)	*p* < 0.05	40.00 (34.00, 57.00)	37.00 (28.00, 49.00)	NS	N/A	N/A	N/A	N/A	N/A	N/A	N/A	N/A	N/A
Maleki Sedgi et al., 2024 [38]	**Rapeseed oil**	27.5 ± 12.1	20.1 ± 6.2	*p* < 0.001	42.7 ± 31.9	28.3 ± 14.3	*p* < 0.001	167.4 ± 42.5	173.6 ± 44.1	*p* = 0.105	N/A	N/A	N/A	N/A	N/A	N/A
Mazloomi et al., 2022 [39]	**Spirulina sauce**	23.13 ± 2.71	18.95 ± 2.72	*p* < 0.001	38.86 ± 4.09	33.25 ± 4.52	*p* < 0.001	43.17 ± 5.73	40.55 ± 4.24	*p* = 0.08	N/A	N/A	N/A	N/A	N/A	N/A
Rafie et al., 2020 [40]	**Ginger powder**	32.69 ± 5.23	31.08 ± 7.85	*p* = 0.312	42.04 ± 8.92	32.21 ± 7.12	*p* < 0.001	N/A	N/A	N/A	N/A	N/A	N/A	FLI: B—89.43 (52.5,97.3); PI—85.21 (39.2,96); *p* < 0.001	Change in the liver fat content after intervention—B: Grade 1–12 (12%); Grade 2–8 (34.78%); Grade 3–3 (13.04%); PI: Grade 1- 13 (56.52%); Grade 2–7 (30.43%); Grade 3–3 (13.04%)	N/A
Soleimani et al., 2020 [43]	**Garlic powder**	48.3 ± 11.6	42.2 ± 11.2	*p* = 0.001	57.8 ± 13.9	47.2 ± 16.1	*p* = 0.001	N/A	N/A	N/A	MASLD progression PI change in hepatic steatosis—improved: 51.1%; unchanged: 46.8%; worsened: 2.1%; *p* < 0.001			N/A	N/A	N/A
Sangouni et al., 2020 [42]	**Garlic powder**	N/A	N/A	N/A	N/A	N/A	N/A	N/A	N/A	N/A	N/A	N/A	N/A	N/A	MASLD stage at B—Mild: 14 (29.7%); Moderate: 29 (61.7%); Severe: 4 (8.6%)	N/A
Sangouni et al., 2020 [41]	**Garlic powder**	22.8± 11.1	20.6 ± 8.6	N/A	30.9± 15.7	26.0± 13.2	N/A	203.8± 56.9	200.3± 49.0	N/A	N/A	N/A	N/A	N/A	B—Grade 1: 11 (24.4%); Grade 2: 29 (64.4%); Grade 3: 5 (11.2%); PI—reduction 1 Grade: 28 (62.2%); reduction 2 Grades: 2 (4.4%); no change: 15 (33.7%); 1 Grade increase: 0 (0%)	N/A

Data is presented as Mean ± SD or Median (25th–75th). B and PI reported as exact values or otherwise marked and displayed as MD. N/A means that there was no data available. AST, ALT, and ALP expressed in IU/L. CAP expressed in dB/m. FLI and grade of fatty liver presented as exact values. *p* value > 0.05 results considered as NS. AST, aspartate aminotransferase; ALT, alanine aminotransferase; ALP, alkaline phosphatase; IHL, intrahepatic lipid; CAP, Controlled Attenuation Parameter; FLI, Fatty Liver Index; HSI, hepatic steatosis index; MD, Mean Difference; SD, Standard Deviation; NS, not significant; N/A, Not Available; B, baseline; PI, post-intervention; fruit-rich diet, FRD; whole-grain products, WGPs; beetroot juice, BJ; green coffee extract, GCE.

**Table 10:** Comparisons of changes in liver function outcomes after interventions with plant-based foods and control/placebo in RCTs conducted in MASLD patients.

Change in Liver Function Outcomes After Interventions with Plant-Based Foods in MASLD							
Dietary Intervention with Plant-Based Food	Hepatic Enzymes			Hepatic Steatosis			Liver Fibrosis
	AST	ALT	ALP	CAP	Hepatic Inflammation (FLI)	Grade of Fatty liver	
FRD [27]	*p* < 0.001 *	*p* < 0.001 *	*p* < 0.001 *	N/A	N/A	N/A	N/A
Whole oranges [28]	*p* = 0.11	*p* = 0.45	*p* = 0.66	*p* < 0.004	N/A	N/A	N/A
WGPs [29]	*p* < 0.001	*p* < 0.001	N/A	N/A	N/A	*p* < 0.001	N/A
HFBs [30]	*p* = 1	*p* = 1	N/A	*p* = 0.04	N/A	N/A	N/A
BJ [31]	*p* = 0.014	*p* < 0.001	*p* < 0.001	N/A	*p* < 0.001	N/A	N/A
GCE [32,44]	*p* = 0.757	*p* = 0.268	N/A	N/A	N/A	N/A	N/A
	*p* = 0.086	*p* = 0.26	N/A	N/A	N/A	*p* = 0.76	N/A
Sour tea [34]	*p* = 0.004	*p* = 0.01	N/A	N/A	N/A	N/A	N/A
Flaxseed oil [35]	*p* = 0.010	*p* = 0.047	*p* < 0.001	N/A	N/A	N/A	N/A
Flaxseed powder [36]	*p* < 0.001	*p* = 0.406	N/A	*p* = 0.276	N/A	N/A	*p* = 0.032
Golden flaxseed powder [37]	*p* = 0.05	NS	N/A	N/A	N/A	N/A	N/A
Rapeseed oil [38]	*p* = 0.119	*p* = 0.051	*p* = 0.004	N/A	N/A	*p* < 0.001	N/A
Spirulina sauce [39]	*p* = 0.02	*p* = 0.03	*p* = 0.70	N/A	N/A	N/A	N/A
Ginger powder [40]	N/A	N/A	N/A	N/A	*p* = 0.116	N/A	N/A
Garlic powder [41,42,43]	*p* = 0.001	*p* = 0.001	N/A	N/A	N/A	N/A	N/A
	N/A	N/A	N/A	N/A	N/A	*p* = 0.29	N/A
	*p* = 0.010	*p* < 0.001	*p* = 0.65	N/A	N/A	*p* = 0.001	N/A
	N/A	N/A	N/A	N/A	N/A	N/A	N/A

Data is presented as a *p* value and color-coded according to the level of significance of the change in reported PI values after interventions with plant-based foods. The *p* values demonstrating a significant increase in the reviewed outcomes are marked with *. N/A means that there was no data available. AST, aspartate aminotransferase; ALT, alanine aminotransferase; ALP, alkaline phosphatase; IHL, intrahepatic lipid; CAP, Controlled Attenuation Parameter; FLI, Fatty Liver Index; HSI, hepatic steatosis index, SD, MD, Mean Difference; SD, Standard Deviation; NS, not significant; N/A, Not Available; B, baseline; PI, post-intervention; fruit-rich diet, FRD; whole-grain products, WGPs; beetroot juice, BJ; green coffee extract, GCE.

## Data Availability

No new data were created or analyzed in this study. Data sharing is not applicable to this article.
